# Determining the key performance indicators of human resource management of military hospital managers; a TOPSIS study

**DOI:** 10.1186/s12875-023-02007-7

**Published:** 2023-02-14

**Authors:** Sadegh Fanaei, Armin Zareiyan, Saeid Shahraki, Abasat Mirzaei

**Affiliations:** 1 Department of Health Services Management, Faculty of Medicine, Aja University of Medical Sciences, Tehran, Iran; 2 Department of Health in Disaster & Emergencies, Nursing Faculty, Aja University of Medical Sciences, Tehran, Iran; 3 Department of Health Care Management, Faculty of Health, Tehran Medical Science, Islamic Azad University, Tehran, Iran

**Keywords:** Human resource management, Key performance indicators, Hospital, TOPSIS

## Abstract

**Background:**

Proper human resource management in military health centers leads to long-term development and improved health-care quality. As a result, the purpose of this research is to identify the key performance indicators of human resource management for military hospital managers, and the unique indicators of military hospitals were obtained.

**Methods:**

This cross-sectional study was performed by the TOPSIS method in the fall of 2021. This study used a checklist consisting of 20 performance indicators of human resource management, which were scored by 20 senior military hospital managers based on two criteria: "importance" and "measurability in military hospitals". The Shannon entropy method was used to weight the indicators, and the BT-TOPSIS Solver software was used to analyze and prioritize them.

**Results:**

Among the 20 indicators in human resource management, the staff satisfaction index in military hospitals, the competitiveness rate of salaries in military hospitals relative to the national sector, the number of permanent staff in military hospitals, and the percentage of contract labor costs in military hospitals have the highest coefficient, respectively.

**Conclusions:**

The importance of human resource management and organizational performance is due to their influence on each other. As a result, human resource management should pay special attention to the professional and personal development of human resources, as this has an impact on the performance of the organization in the long run. In light of the sensitive nature of human resource management and its crucial role in achieving any organization's strategic goals, selecting appropriate indicators is essential.

The Department of Military Health requires indicators unique to the military sector to assess the human resource management of the military hospital, since according to the existing circumstances, the indicators of the military sector and those of the civilian sector are different.

## Background

Hospitals are complex and multi-purpose organizations that use the latest technologies. The management of health care organizations using performance data accelerated in the late twentieth century [1].

With thorough planning, we may make it easier to attain objectives if we treat the hospital organization like a machine and have clear expectations of it. Without taking into account the hospital's numerous sub-systems, the facility's operations will be disrupted [2, 3]. Organizational complexity, high costs, and specialization of healthcare services have increased the importance of performance evaluation in healthcare organizations while also making it easier to achieve goals through performance evaluation [1, 4].

Therefore, health organizations need a clear framework for sustainable development evaluation of human resource management performance. To evaluate the performance of hospital human resource management, high-performance and precision tools are needed so that health managers can select and implement appropriate strategies to improve the performance of their human resources based on the evaluation results [5, 6].

The challenges of human resources in the field of health are more severe in low and middle income countries than in other developed countries and the shortage of skilled labor in less developed countries such as Nigeria has created many problems for people to access standard services [7–9]. Unfair distribution of manpower has resulted in very few human resources present in remote rural areas; the Absence of employees and low motivation, low salaries, poor support, and poor working conditions exacerbate these challenges [10, 11]. We must create synergies in the key skills of human resource managers to address the challenges of human resources in this area [12]. Manpower problems are recognized as the primary barriers to providing quality health care. For this purpose, solutions to manpower problems must be considered in health care systems [13].

The heads of the health care system are obliged to provide effective and fair health care to do this; they need evidence and studies to identify and select policies in this field to have the best performance [14]. To identify key issues related to human resources of health, a series of frameworks have been defined, and studies on improving the performance of human resources have been reviewed. However, many policies have not been reviewed. The key performance indicator allows managers to use this tool as a lever in the management dashboard to improve organizational performance. In today's world, these indicators are essential for planning and control [15]. Labor costs typically make up 75% of a hospital budget, and the cost of health care, in general, is increasing both globally and as a ratio of GDP [16]. Although the importance of determining human resource management indicators in the health system is increasingly understood, it is still difficult to achieve new results from these indicators. Human Resource Management Indicators for attracting, directing, and retaining an efficient workforce is a tool that determines the degree of alignment and appropriateness of strategic goals with human resource management goals [17]. The uses of the HR index are understood by hospitals that seek to develop within the organization at a low cost. If the health care organization spends energy on developing its human resources, it can develop things, increase staff satisfaction, and achieve goals faster at lower costs than other methods [18–21]. As Nefri etal showed, there is a significant positive relationship between human resource strategies and organizational performance in the hospital [22] And success in human resource management requires developing and implementing appropriate human resource strategies [23, 24].

In the military sector, the situation will be more complex and different strategies will be used under specific circumstances, and even a reduction in manpower may be chosen as an appropriate solution [25]. There are special conditions in military systems. The importance of manpower in the military health sector is a key asset [26] that having the general conditions of manpower in the health system requires special security and physical competencies. To this end, creating promotion opportunities, attractive payment methods, and training opportunities can improve the human resource status of the military medicine field [25].

Determining the performance indicators of military hospital managers and using these indicators in the management of these hospitals can pave the way for achieving the security and health goals of the Armed Forces. Manpower is the most important field of management in any hospital. Therefore, in this study, we decided to examine the key performance indicators of manpower management in military hospitals.

Several methods can be used to determine these indicators. Studies have shown that the TOPSIS method is used the most, and about 30% in studies [27]. For this reason, in this study, we decided to determine the key performance indicators of military hospital managers in Tehran by the TOPSIS method.

## Methods

### Study design and recruitment

**Fig. 1:**
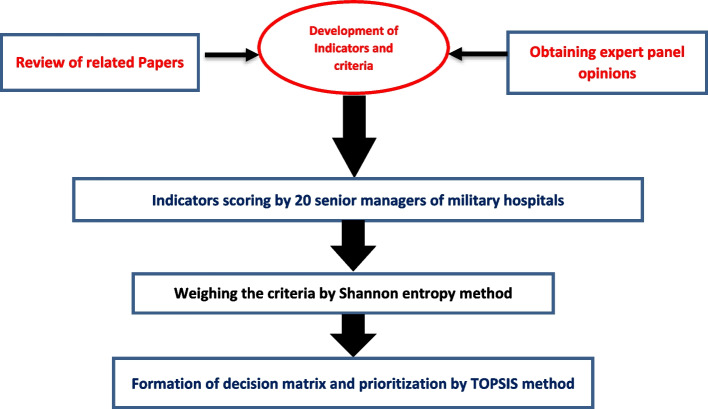
Summery of steps in conducting research

The study was performed from September to December 2021. In terms of time, this study is a cross-sectional study and in terms of methodology, it is a multi-criteria decision-making method. The study population was senior managers of military hospitals. Convenience sampling was used as the sample technique in this study. In this way, twenty sample were selected by snowball method and purposefully with maximum diversity. The process of this study is summarized in Fig. 1.

### Make-up measurement instrument

The measurement instrument of the study was a checklist containing performance indicators of human resource management [6]. To make-up these indicators, first, by reviewing 30 related articles, 80 performance indicators in human resource management were extracted. Then the three-step Delphi method was used. In the first stage, a panel of experts consisting of 8 experts examined 80 indicators (items) extracted from the literature review and gave a scored from 1 to 10. The items that received a score less than 3 (38 indicators) were removed and the items that received a score higher than 7 (6 indicators) were selected, and other items (36 indicators) advanced to the second stage. These 36 indicators were re-examined in the second stage, and scoring was done from 1 to 10. Fourteen indicators received a score above 7 and the remaining score of 7 and below were obtained and eliminated. Thus, these 14 indicators and 6 indicators of the first stage were a total of 20 selected indicators from the first and second stages, which entered the third stage.

### Data analysis

According to experts, the two criteria of "importance" and "measurability in military hospitals" were considered to prioritize the indicators.

In the third stage, in order to prioritize the indicators and determine the key indicators of human resource management performance in military hospitals, 20 finalized indicators were given to 20 experts. These experts were selected non-randomly based on their experience in managing military hospitals. An attempt was made to include maximum variety in the selection of samples.

After scoring the indices by the 20 experts, the Shannon entropy method was used to weight the indices. Finally, BT-Topsis Solver software was used to analyze and prioritize them. The steps of conducting research are shown in the diagram below.

## Results

TOPSIS is one of the methods of the Multi Criteria Decision Making (MCDM) model proposed by Huang and Eun in 1981 [28]. In this method, the *m* options are evaluated by *n* criteria. The agreed principle of the TOPSIS method for multi-criteria decision making is that the selected options should have the shortest distance from the positive ideal solution and the maximum distance from the negative ideal solution [28]. It is a method of compensatory aggregation that compares a set of alternatives, normalizing scores for each criterion and calculating the geometric distance between each alternative and the ideal alternative, which is the best score in each criterion. An assumption of TOPSIS is that the criteria are monotonically increasing or decreasing. Normalization is usually required as the parameters or criteria are often of incongruous dimensions in multi-criteria problems [27].

In this paper, the purpose of applying the TOPSIS method is to use the concept of positive and negative ideal options so that each option is extracted based on the criteria set to determine the key performance indicators of senior managers of military hospitals in the field of human resource management so that the functional options have the closest distance to the positive ideal values and the farthest distance to the ideal negative point. Twenty participants were selected based on their managerial experience in military hospitals. The sampling method was non-random and it was done by the snowball sampling method with maximum diversity in the selection of samples [29–31]. Table 1 shows the characteristics of 20 experts who prioritized the selected indicators. The mean age of respondents was 46.95 years and the mean of managerial experience years was 10.35 years. All experts in this study were male.

**Table 1:** Demographic characteristics of respondents (*n* = 20)

Experts	Age	Managerial Experience/Years	Gender
1	45	8	**Male**
2	44	9	**Male**
3	48	10	**Male**
4	45	10	**Male**
5	49	12	**Male**
6	52	14	**Male**
7	52	15	**Male**
8	43	8	**Male**
9	48	9	**Male**
10	50	11	**Male**
11	43	7	**Male**
12	47	8	**Male**
13	49	8	**Male**
14	48	8	**Male**
15	47	9	**Male**
16	45	15	**Male**
17	47	13	**Male**
18	48	12	**Male**
19	44	11	**Male**
20	45	10	**Male**

### Steps of performing TOPSIS method

#### Decision matrix formation

The first step was to create a decision matrix that includes a set of criteria and options. The criterion is placed in columns in this matrix, and the options are placed in rows. The criterion means two criteria of importance and ability to measure the key indicators of human resource management performance in military hospitals and options include 20 indicators selected by experts to determine key performance indicators. Each matrix cell evaluates each option against each criterion. After the decision matrix was formed, using the Likert scale from 1 to 10, the opinions of 20 experts were obtained, and then their average opinions were formed in the form of a decision matrix (Table 2).

**Table 2:** Expert’s opinions average matrix

*Options*	*Indicators Content*^*♠*^	*Importance*	*Measurability*
*1*	Percentage of contract labor costs in military hospitals	7.3	8.8
*2*	The rate of competitiveness of military hospitals' salaries compared to the national sector	8.4	8.2
*3*	Staff satisfaction index in military hospitals	8.65	8.15
*4*	Percentage of administrators' leave used in military hospitals	5.7	8.15
*5*	Absence and telecommuting rates in military hospitals	6.4	8.15
*6*	The turnover of staff jobs in military hospitals	7.4	7.8
*7*	Number of official staff in military hospitals	7	9.8
*8*	Number of contract staff in military hospitals	5.65	9.7
*9*	The average service life of contract staff in military hospitals	6.5	8.45
*10*	The cost of office space per employee in a military hospital	5.3	6.6
*11*	The level of loyalty of employees to the organization in military hospitals	8.6	6.7
*12*	Staff productivity rates in military hospitals	8.75	6.95
*13*	Amount of staff suggestions implemented in military hospitals	6.6	5.55
*14*	Percentage of substandard labor performance in military hospitals	6.9	6.55
*15*	Costs for recruiting any new staff in military hospitals	6.55	7.05
*16*	The average time of filling a vacancy in the organizational structure of military hospitals	6.25	6.5
*17*	The attractiveness of the organization among the manpower outside the military hospitals	7.85	6.25
*18*	Percentage of applicants for employment in military hospitals	6.6	5.85
*19*	Rates of conversion of contracted manpower into formal military hospitals	6.75	7.2
*20*	Percentage of recruitment of personnel introduced through the current staff of military hospitals	5.05	6.3
*Criterion Type*		POSITIVE	POSITIVE
*Criterion Weight*		0.4912	0.5088

♠The content of this column is actually the same as the decision matrix options

#### Normalizing the decision matrix

Matrix normalization was done that each value was divided by the square root of the sum of the values of that column. In this step, the decision matrix becomes a normalized matrix (Table 3).

**Table 3:** Normalized matrix

*Options*^*♣*^	*Importance*	*Measurability*
*1*	0.233	0.261
*2*	0.268	0.243
*3*	0.276	0.242
*4*	0.182	0.242
*5*	0.204	0.242
*6*	0.236	0.231
*7*	0.223	0.291
*8*	0.180	0.288
*9*	0.207	0.251
*10*	0.169	0.196
*11*	0.275	0.199
*12*	0.279	0.206
*13*	0.211	0.164
*14*	0.220	0.194
*15*	0.209	0.209
*16*	0.199	0.193
*17*	0.251	0.185
*18*	0.211	0.173
*19*	0.215	0.213
*20*	0.161	0.187

^♣^The content of the options column is the same as the indicators in Table 1

#### Create A weighted normalized matrix

In this step, the weights of the criteria obtained from Shannon Entropy method were multiplied by the normal matrix to obtain the weighted matrix. The weight obtained from Shannon Entropy method was calculated automatically by the software (Table 4).

**Table 4:** Normalized matrix weighting

*Options*^*♣*^	*Importance*	*Measurability*
*1*	0.114	0.133
*2*	0.131	0.123
*3*	0.135	0.123
*4*	0.089	0.123
*5*	0.100	0.123
*6*	0.116	0.117
*7*	0.109	0.148
*8*	0.088	0.146
*9*	0.102	0.127
*10*	0.083	0.099
*11*	0.135	0.101
*12*	0.137	0.105
*13*	0.103	0.083
*14*	0.108	0.099
*15*	0.102	0.106
*16*	0.098	0.098
*17*	0.123	0.094
*18*	0.103	0.088
*19*	0.106	0.108
*20*	0.079	0.095

^♣^The content of the options column is the same as the indicators in Table 1

#### Finding the ideal and counter-ideal solution

The type of criteria must be specified. The criteria are either positive or negative. In this study, two criteria were considered positive criteria, the increase of which improves the system. Given that the criteria are positive, the positive ideal was considered the largest value of that criterion and the negative ideal was considered the smallest value of that criterion (Table 5).

**Table 5:** Determining the solution of positive ideal and negative ideal

*Optimal Solution*	Importance	Measurability
*Positive (* +*)*	0.137	0.148
*Negative (-)*	0.079	0.083

#### Calculate the distance from the ideal and counter-ideal solution

At this step, the distance of each option from its positive and negative ideals was calculated and then the distance between each option was measured. That is, the distance between the options and the ideal positive and negative options was determined. The result is shown in Table 6.

**Table 6:** Distance size from the positive and negative ideal solution

*Options*^*♣*^	*Positive*	*Negative*
*1*	0.027	0.060
*2*	0.024	0.066
*3*	0.025	0.068
*4*	0.054	0.040
*5*	0.044	0.044
*6*	0.036	0.050
*7*	0.027	0.071
*8*	0.048	0.063
*9*	0.040	0.049
*10*	0.072	0.016
*11*	0.046	0.058
*12*	0.043	0.061
*13*	0.072	0.024
*14*	0.057	0.032
*15*	0.054	0.032
*16*	0.063	0.023
*17*	0.055	0.045
*18*	0.068	0.024
*19*	0.050	0.036
*20*	0.078	0.011

^♣^The content of the options column is the same as the indicators in Table 1

#### Calculation of similarity index and ranking of options

The similarity index indicates the score of each option. The closer this index is to the number one, the better it is Table 7

**Table 7:** The ranking of indicators (Options)

*Options*	*Indicators Ranking*	*Proximity Coefficient*
*3*	Staff satisfaction index in military hospitals	0.733
*2*	The rate of competitiveness of military hospitals' salaries compared to the national sector	0.727
*7*	Number of official staff in military hospitals	0.721
*1*	Percentage of contract labor costs in military hospitals	0.688
*12*	Staff productivity rates in military hospitals	0.589
*6*	The turnover of staff jobs in military hospitals	0.576
*8*	Number of contract staff in military hospitals	0.565
*11*	The level of loyalty of employees to the organization in military hospitals	0.554
*9*	The average service life of contract staff in military hospitals	0.547
*5*	Absence and telecommuting rates in military hospitals	0.500
*17*	The attractiveness of the organization among the manpower outside the military hospitals	0.449
*4*	Percentage of administrators' leave used in military hospitals	0.429
*19*	Rates of conversion of contracted manpower into formal military hospitals	0.420
*15*	Costs for recruiting any new staff in military hospitals	0.376
*14*	Percentage of substandard labor performance in military hospitals	0.364
*16*	The average time of filling a vacancy in the organizational structure of military hospitals	0.271
*18*	Percentage of applicants for employment in military hospitals	0.265
*13*	Amount of staff suggestions implemented in military hospitals	0.251
*10*	The cost of office space per employee in a military hospital	0.183
^*20*^	^Percentage of recruitment of personnel introduced through the current staff of military hospitals^	^0.126^

^*^Green rows are the most important indicator and pink rows are the least important

## Discussion

Finding the important performance indicators of managers, specifically for military hospitals, was our aim in this study. To be superior to other competitors, large and small hospitals need to examine the performance of senior managers based on key indicators in the field of human resources so that the performance appraisal process is commensurate. According to various studies, there is a significant relationship between human resource management and organizational performance [32–34]. Therefore, in human resource management, attention should be paid to the job and personal development skills of human resources because in the long time it will affect the performance of the hospital.

In the past, we have not achieved specialized results in this field to identify key performance indicators of human resource management of senior managers of military hospitals, and so far, no specific research has been conducted for this purpose in military organizations.

According to the results of the present study, the indicators of "Staff Satisfaction in Military Hospitals", Competitiveness rate of salaries of military hospitals compared to the national sector", Number of official staff in military hospitals" and "Percentage of contract labor in Military hospital" had the highest rank, respectively.

The highest score among all indicators of this study belonged to the index of "staff satisfaction in military hospitals" And this indicator determines how senior managers of military hospitals work. Also in a study by Quinto et al. Conducted at the Uganda Military Hospital, results showed that low motivation among the hospital's manpower led to increasing in job dissatisfaction. And senior managers of the military health system of this country to improve the hospital's performance should use various methods to increase the satisfaction of manpower [35]. Staff awareness and perception of the performance of senior managers of military hospitals will have a significant effect on job satisfaction of military hospital staff. Different dimensions of job satisfaction of military hospitals will be effect on improving the performance of manpower [36]. Due to the interference and occupational conflicts of the health sector with military restrictions, senior managers of military hospitals should continuously evaluate and improve the satisfaction of manpower [35]. Low job satisfaction in Iranian military hospitals can pose many risks to the country's security.

Significant differences in the salaries and benefits of human resources in military hospitals compared to other organizations and civilian hospitals in the country have caused functional problems in the management system of military hospital managers [37]. In this regard, the results of a study by Bahadori et al. In a military hospital showed that most of the staff productivity index scores were less than good. One of the most important factors influencing these results has been the lower salaries of military hospitals than other health organizations in the country. Numerous studies in low-income countries have shown that salaries based on manpower performance improve the quality of hospital performance compared to traditional payment methods [38, 39]. Also, the study of Doo et al. Showed that the health care department should compare and evaluate the salaries and incomes of physicians in public hospitals with the salaries and incomes of other industries and disciplines and their colleagues in other hospitals to understand the correct way to pay salaries. And Salary and fringe benefits to human resources [40]. Implementing the amended tariffs will redistribute the costs allocated to manpower, which will create a more reasonable proportion of revenues [41].

Most of the resources and funds of the healthcare system are allocated to manpower. Because of the special conditions that exist in military hospitals, especially in each country's army, a large amount of manpower is made up of official personnel. For some of the needs of the hospital, contract military and part-time force is used by conducting special military interviews. In order to successfully complete their missions, Military hospitals need more than standard manpower to carry out the special missions assigned to them. In military health organizations, like other organizations, employee commitment is important. In this regard, loyalty and belonging of manpower to the hospital can affect the organizational performance of the hospital [42]. Also, the results of the study of Demir et al. In the military sector show that professional commitment and organizational motivations contribute to organizational commitment, while the relationship between individual conflict and organizational goals has been significant [35].

In military hospitals, the basic salary and insurance of the official forces are paid by higher organizations and in most cases, manpower incentives and facilities are paid for by the hospital. However, if the hospital fails to meet its needs with its official manpower, it will seek to recruit contractual Forces. In some cases, due to the managerial performance of the military hospital, the number of contracted personnel is more than the actual need and puts much financial burden on the hospital.

## Conclusion

The efficiency of the military hospital will increase if the official forces of the military hospital are properly managed and a suitable payment model is defined based on their performance instead of the traditional military payment. In this way, senior managers value employees and show that employees' efforts to achieve organizational goals have been considered [43]. Line managers are heavily involved in medical personnel management in hospitals and human resource management systems may also increase the quality of managers [44, 45].

Finally, considering the importance of the health care system and military hospitals in the country and the lack of studies and specialized results on key performance indicators and how to manage these hospitals, it is suggested about other areas of military hospital management and compare it with these indicators should also be studied in civilian hospitals. Obviously, after determining these indicators, we can plan to improve the quality of work of military hospitals with a more open vision and more accurate information. 

## Limitations

Due to the military nature of the research filed, it was not possible to access all senior managers. The COVID-19 epidemic at the time of the research was another limitation of this study.

## Data Availability

Due to conducting research in the military environment, it is not possible to provide data in public, but in case of correspondence with the corresponding author, data and material will be provided.

## Abbreviations

MCDM: Multi Criteria Decision Making
HRM: Human Resource Management
TOPSIS: Technique for Order of Preference by Similarity to Ideal Solution
